# A TLR5 mono-agonist restores inhibited immune responses to *Streptococcus pneumoniae* during influenza virus infection in human monocytes

**DOI:** 10.1371/journal.pone.0258261

**Published:** 2021-10-13

**Authors:** Paula T. Maguire, Sinéad T. Loughran, Ruth Harvey, Patricia A. Johnson

**Affiliations:** 1 Viral Immunology Laboratory, School of Nursing, Psychotherapy and Community Health, Dublin City University, Dublin, Ireland; 2 Department of Applied Science, Dundalk Institute of Technology, County Louth, Ireland; 3 National Institute for Biological Standards and Controls, Potters Bar, Herts, United Kingdom; University of South Dakota, UNITED STATES

## Abstract

Influenza A virus (IAV) predisposes individuals to often more severe secondary bacterial infections with *Streptococcus pneumonia* (*S*. *pneumoniae*). The outcomes of these infections may be made worse with the increase in antimicrobial resistance and a lack of new treatments to combat this. Th17 responses are crucial in clearing *S*. *pneumoniae* from the lung. We previously demonstrated that early IAV infection of human monocytes significantly reduced levels of *S*. *pneumoniae*-driven cytokines involved in the Th17 response. Here, we have further identified that IAV targets specific TLRs (TLR2, TLR4, TLR9) involved in sensing *S*. *pneumoniae* infection resulting, in a reduction in TLR agonist-induced IL-23 and TGF-β. The effect of IAV is more profound on the TLR2 and TLR9 pathways. We have established that IAV-mediated inhibition of TLR9-induction is related to a downregulation of RORC, a Th17 specific transcription factor. Other studies using mouse models demonstrated that TLR5 agonism improved the efficacy of antibiotics in the treatment of IAV/*S*. *pneumoniae* co-infections. Therefore, we investigated if TLR5 agonism could restore inhibited Th17 responses in human monocytes. Levels of pneumococcus-driven cytokines, which had previously been inhibited by IAV were not reduced in the presence of the TLR5 mono-agonist, suggesting that such treatment may overcome IAV inhibition of Th17 responses. The importance of our research is in demonstrating the IAV directly targets *S*. *pneumoniae*-associated TLR pathways. Additionally, the IAV-inhibition of Th17 responses can be restored by TLR5 agonism, which indicates that there may be a different Th17 signalling pathway which is not affected by IAV infection.

## Introduction

Annually, influenza A virus (IAV) causes the deaths of hundreds of thousands of individuals, and most of these deaths can be attributed to secondary bacterial infections [1–3]. Commonly, these secondary bacterial infections are caused by *Streptococcus pneumoniae* (*S*. *pneumoniae*) [4–7]. Infections such as these affect both patients with pre-existing conditions, and previously healthy individuals [8–10]. Additionally, the number of deaths due to such co-infections is likely to increase due to the continuing emergence of antimicrobial resistant strains of bacteria [11]. A more complete understanding of the mechanisms that lead to susceptibility to bacterial disease during IAV infection is required in order to treat these infections effectively.

Th17 cells have been identified as critical in the clearance of extracellular bacteria such as *S*. *pneumoniae* from the lung and further studies have revealed that IAV infection has been shown to inhibit the Th17 response in mice [12,13]. We have previously demonstrated that IAV directly targets individual cytokines in the Th17 response to *S*. *pneumoniae*, specifically IL-23 in primary human monocytes [14].

Immune cells recognise pathogens such as *S*. *pneumoniae* through receptors such as TLRs, and NOD-like receptors (NLRs) [15]. Activation of TLRs triggers signalling cascades, which drive differentiation of cytokine expression and ultimately Th cell polarisation. It has been established that the TLRs involved in sensing *S*. *pneumoniae* infection are TLR2, TLR4, and TLR9 [16]. We hypothesised that IAV may be directly targeting individual *S*. *pneumoniae*-associated TLRs. Therefore, in this study, we examined the effects of IAV infection on TLR mono-agonist induction of cytokines involved in the Th17 response in primary human monocytes. Furthermore, retinoic acid receptor (RAR)-related orphan receptor C (RORC), has been identified as a Th17-specific transcription factor, which has a very specific role in the development of Th17 cells [17,18]. RORC is the gene in humans which encodes for two protein isoforms: RAR-related orphan receptor gamma (RORγ) and RORγt, previously known as RORC1 and RORC2, respectively [19]. RORγt, which is exclusively expressed in a variety of immune cells, is a transcription factor, which has been identified as a potential master regulator for driving Th17 cell differentiation in both mice and humans [17,18,20–24]. Expression of RORγt is induced by TGF-β and IL-6 or IL-21 in a STAT3-dependent manner in humans and mice [25–27], which is key for the expression of IL-17 [17]. The role of RORγ remains to be controversial as there are contradictions regarding cell type expression [28–32]. In mice, an additional transcription factor, RORα has also been implicated in Th17 differentiation [33], however the influence of RORα in humans appears to be weaker [28]. RORγt also plays an important role in the upregulation of IL- 23 [17]. As RORC has previously been detected in human monocytes [32], we also sought to determine if IAV was directly targeting RORC expression. In addition, previous work in mouse models have established that a TLR5 agonist can have beneficial therapeutic effects when combined with antibiotic administration in the treatment of IAV and *S*. *pneumoniae* co-infections [34]. Although, the exact mechanisms behind these improved outcomes have not been fully elucidated [34]. Therefore, we have endeavoured to determine whether a TLR5 mono-agonist could restore IAV-inhibited immune responses to *S*. *pneumoniae* in primary human monocytes [14]. In this study, our findings demonstrate that IAV targets each of the *S*. *pneumoniae* associated TLR pathways. TLR-induced IL-23 and TGF-β were inhibited by IAV, however the level of inhibition was greater following TLR2 and TLR9 agonism. Additionally, inhibition of TLR9-induced IL-23 correlates with downward pressure on RORC by IAV infection. Finally, TLR5 mono-agonism circumvents IAV-inhibition of immune responses to *S*. *pneumoniae* These studies further illuminate the mechanisms behind IAV inhibition of Th17 responses.

## Materials and methods

### Ethical approval

Ethical approval for this study was granted by Dublin City University’s Research Ethics Committee.

#### Separation of PBMCs

Buffy coats from healthy donors were obtained from the Irish Blood Transfusion service. Peripheral venous blood was mixed with 5% EDTA in 1X PBS and diluted 1:2 with HBSS. Diluted blood was layered onto density gradient medium Lymphoprep^TM^ (Axis-shield, Norway) and centrifuged at 400xg for 25 minutes. The buffy coat layer was removed and cells were washed with HBSS and resuspended in complete RPMI (cRPMI).

#### Separation of CD14^+^ monocytes

PBMCs were resuspended in MACs buffer and incubated with CD14^+^ microbeads. Cells were washed with MACs buffer and cell suspension was applied to an LS column attached to a magnet. Positively labelled CD14^+^ cells were flushed out of the column with a plunger in the absence of magnetic force. CD14^+^ cells were centrifuged at 800xg for 5 minutes and resuspended in cRPMI at a density of 1x10^6^ cells/ml.

#### Viruses and infections

Isolated CD14^+^ cells at a density of 10^6^ cells/ml were infected with live H1N1 (Puerto-Rico/8/34) or H3N2 (A/Uruguay/716/2007) (4.9 PFU/ml and MOI 2) for 2 hours, centrifuged at 3,000xg for 5 minutes and resuspended in fresh cRPMI. Subsequently, infected cells were incubated alone or in combination with Heat Killed *S*. *pneumoniae* (HKSP) (10^7^ CFU) (Invivogen), or TLR agonists for 24 h.

#### TLR stimulations

TLR mono-agonists were purchased from Invivogen and were mono-stimulants. Lipoteichoic acid from *Staphylococcus aureus* (LTA-SA) (20 μg) was used as a TLR2 mono-agonist. Ultra-pure LPS from *E*. *coli* (LPS-EB) (100 ng) was used a TLR4 mono-agonist. Class A CpG oligonucleotide (ODN 2216) (2 μM) was used as a TLR9 mono-agonist. Flagellin from *Salmonella typhimurium* (FLA-ST) (100 ng) was used as TLR5 mono-agonist. Isolated CD14^+^ cells at a density of 10^6^ cells/ml were stimulated with mono-agonists at chosen doses and incubated for 24 h at 37°C.

#### ELISA

Supernatant from treated cells was used to detect for the following cytokines; IL-23 and IL-12p70 (BioSciences), IL-6, IL-1β, IL-27, TGF-β, IL-10 (R&D Systems) according to manufacturer’s protocol. At least 5 experimental repeats of each treatment were analysed for each cytokine/donor.

#### qPCR

Total RNA was isolated by using the RNeasy Plus mini kit (Qiagen). Total RNA was reverse transcribed into cDNA using the GoScript Reverse Transcription System (Promega). cDNA samples were amplified using the Lightcycler Nano (Roche Diagnostics) and Faststart Essential DNA Probes Master System (Roche Diagnostics) with RealTime Ready Assays (Roche Diagnostics) or Universal Probes (Sigma Aldrich). Expression of RORC was normalised to the expression of the reference gene, GAPDH. The chosen RORC assay (Assay ID: 102571) detected both variants of RORC (ROR-γ and ROR-γ). The fold change in gene expression was calculated using the equation: 2^(-ΔΔCq)^. The primer sequences for nucleoprotein (NP) were as follows: H1N1 NP forward: GGTGCTGCAGTCAAAGGAGT; reverse: CCCACGTTTGATCATTCTGA; H3N2 NP forward: GGTGCTGCAGTCAAAGGAAT; H3N2 NP reverse: CCCCGTTTGACCATTCTG. NP primers were used with a universal probe (4694414001).

#### Statistical analysis

Statistical analyses were performed using GraphPad Prism version 6.0 for Mac (GraphPad Software). Data was normalised by setting untreated sample readings to ~1 and comparing treated sample readings to that value, thus providing relative concentrations. Data has been normalised to allow for the presentation of data for multiple human donors; this is due to donor-to-donor variability in relation to actual concentration of cytokines produced. A One-Way ANOVA was fitted to the data and comparisons of interest were made using a Sidak test to adjust for multiple testing, using a 5% significance interval; p-values less than 0.05 were considered significant and are represented as follows: *p<0.05, **p<0.01, ***p<0.001, ****p<0.0001.

## Results

### Live influenza A virus infection is reproducible in human monocytes

**Fig 1:**
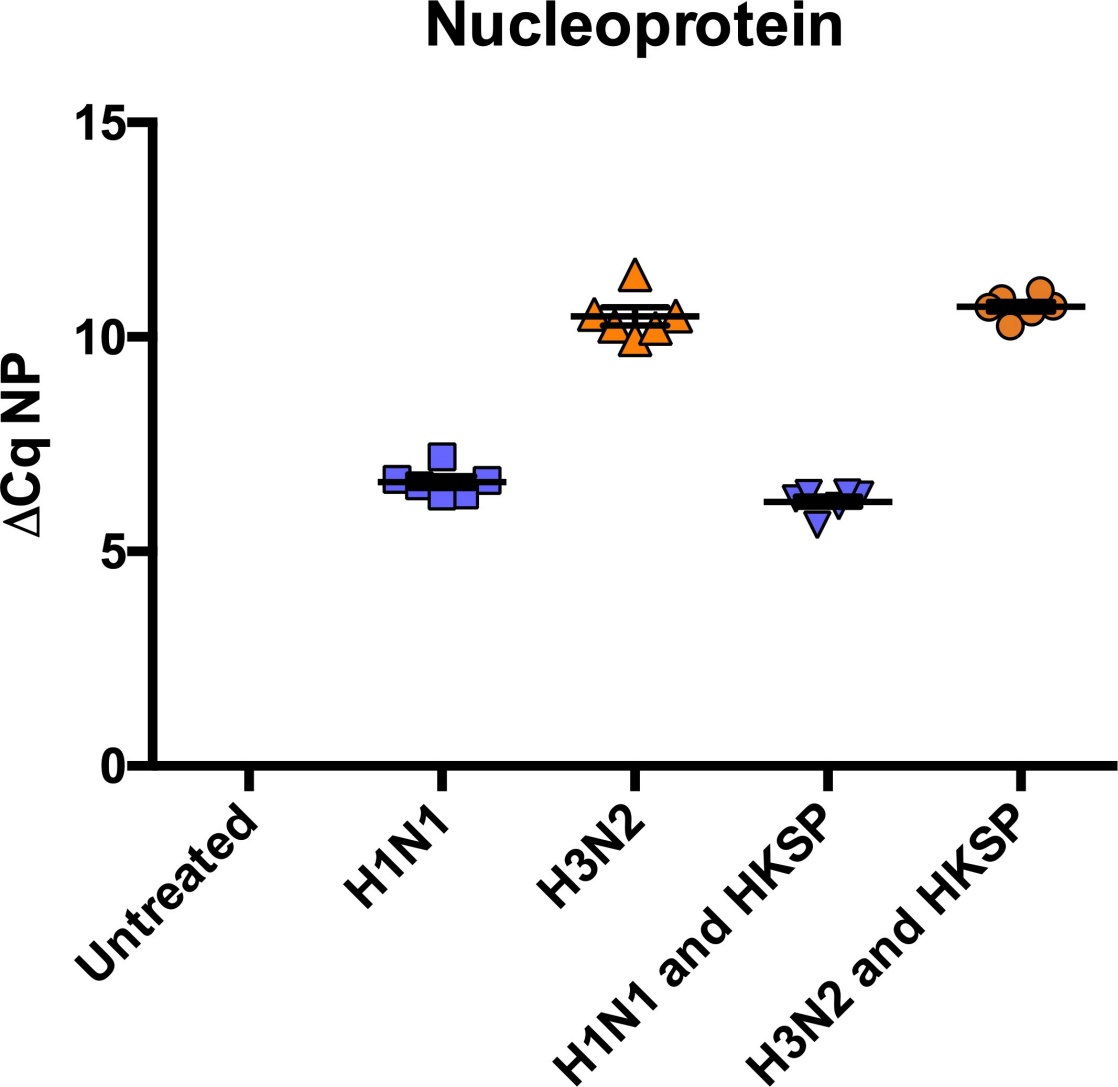
H1N1 and H3N2 are confirmed to infect human monocytes. The levels of H1N1 and H3N2 NP mRNA expression by human monocytes following 24 hr treatment with live H1N1 or H3N2 alone or in combination with HKSP or untreated as a control were determined by qPCR. Cq values were normalised to the expression of GAPDH and plotted on a scatter dot plot, where higher Cq values indicates lower abundance. Each range of dots represents normalised expression of NP + SEM of 2 experimental repeats of each treatment in the same donor (n = 3). Fold expression was not calculated as NP was not amplified in untreated samples.

The influenza nucleoprotein (NP) is a highly conserved viral protein, which is essential for viral replication. As NP is expressed intracellularly during influenza infection, it can serve as an indicator to confirm IAV infection [35,36]. No NP (H1N1 or H3N2) was detected in untreated cells. H1N1 NP was detected in H1N1-infected cells alone or in combination with HKSP (Fig 1). H3N2 NP was detected in H3N2-infected cells alone or in combination with HKSP (Fig 1). Expression of NP was very consistent between H1N1-infected donors and H3N2-infected donors irrespective of HKSP treatment, although higher levels of NP expression was detected in H1N1- infected donors.

### Influenza A virus infection specifically targets TLR2 pathway in human monocytes

**Fig 2:**
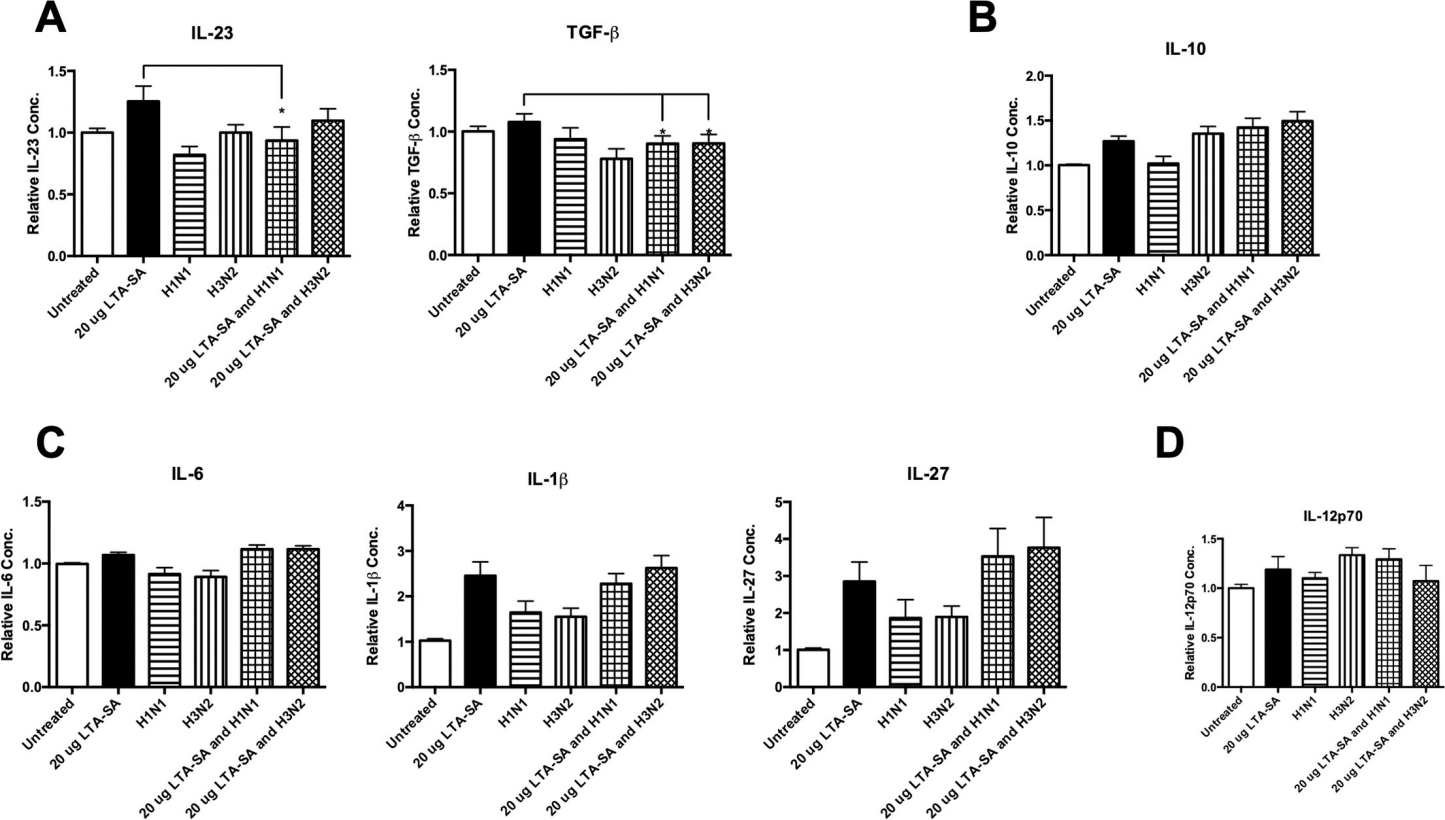
Live IAV specifically targets TLR2 pathway in human monocytes. Levels of (A) IL-23, TGF-β, (B) IL-10, (C) IL-6, IL-1β, IL-27, and (D) IL-12p70 secreted by CD14^+^ cells following 24 h treatment with a TLR2 mono-agonist (LTA-SA), live H1N1 or H3N2 alone or in combination with LTA-SA (or untreated as a control) were determined by ELISA. Each column represents normalised mean cytokine levels + SEM of 3 technical repeats of each treatment in every donor. Each ELISA represents normalised results from multiple donors (n): (A) IL-23 (n = 9), TGF-β (n = 9), (B) IL-10 (n = 9), (C) IL-6 (n = 9), IL-1β (n = 9), IL-27 (n = 9), and IL-12p70 (n = 5). Statistical analyses were performed to compare cytokine levels in cells treated with LTA-SA versus cells exposed to live H1N1 or H3N2 in combination with LTA-SA by fitting a One-Way ANOVA to the data and using a Sidak test to adjust for multiple testing (*p<0.05).

It has been demonstrated in mice that in the absence of TLR2 signalling, transmission of *S*. *pneumoniae* occurred more efficiently during co-infection with influenza [37]. Both human and mouse models have shown that TLR2 senses *S*. *pneumoniae* infections [38–40]. We sought to establish if IAV had an effect on TLR2 agonsim in human monocytes. We have demonstrated that primary human monocytes respond to LTA-SA stimulation and that IAV infection inhibits these responses 24 hours post-infection. We found that the H1N1 subtype of IAV significantly inhibited LTA-SA induced Th17 polarising IL-23 (n = 9), whereas both subtypes of IAV significantly inhibited LTA-SA induced Th17 polarising TGF-β (n = 9) (Fig 2A). There was no increase in the anti-inflammatory cytokine, IL-10 from cells co-treated with LTA-SA and IAV (n = 9) (Fig 2B). There were no significant effects on the Th17 polarising cytokines, IL-6 and IL-1β (n = 9), or on the multi-functional cytokine, IL-27 (n = 9) (Fig 2C). There was also no effect on the Th1 polarising cytokine, IL-12p70 (n = 5) (Fig 2D).

### Influenza A virus infection inhibits TLR4 mono-agonist-induced TGF-β in human monocytes

**Fig 3:**
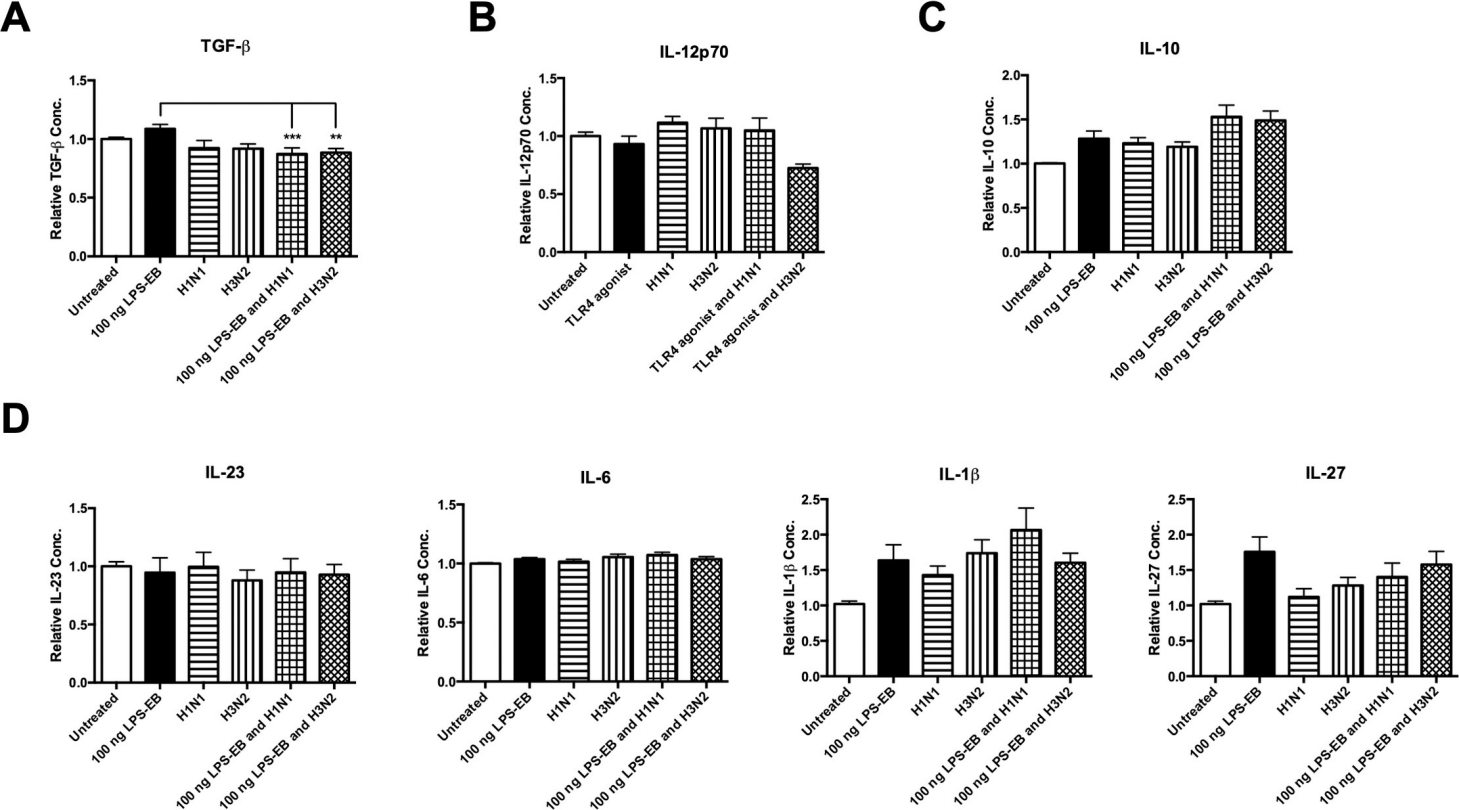
Live IAV targets TLR4-induced TGF-β in human monocytes. Levels of (A) TGF-β, (B) IL-12p70, (C) IL-10, (D) IL-23, IL-6, IL-1β, and IL-27 secreted by CD14^+^ cells following 24 h treatment with a TLR4 mono-agonist (LPS-EB), live H1N1 or H3N2 alone or in combination with LPS-EB (or untreated as a control) were determined by ELISA. Each column represents normalised mean cytokine levels + SEM of 3 technical repeats of each treatment in every donor. Each ELISA represents normalised results from multiple donors (n): (A) TGF-β (n = 9), (B) IL-10 (n = 9), (C) IL-12p70 (n = 5), (D) IL-23 (n = 9), IL-6 (n = 9), IL-1β (n = 9), and IL-27 (n = 9). Statistical analyses were performed to compare cytokine levels in cells treated with LPS-EB versus cells exposed to live H1N1 or H3N2 in combination with LPS-EB by fitting a One-Way ANOVA to the data and using a Sidak test to adjust for multiple testing (**p<0.01, ***p<0.001).

Studies have shown that TLR4 is triggered in response to *S*. *pneumoniae* infection by recognising PLY in both human cell lines and mice [41–43]. We identified that primary human monocytes also respond to the TLR4 mono-agonist, although not as strongly and that IAV inhibits the LPS-EB induction of the multi-functional cytokine, TGF-β in these cells (n = 9) (Fig 3A). Basal induction of the Th1 cytokine, IL-12p70 (n = 5) (Fig 3B) was reduced by H3N2, although not significantly. There was a slight increase in anti-inflammatory IL-10 (n = 9) (Fig 3C) from cells treated with LPS-EB and IAV, although this increase was also not statistically significant. LPS-EB and basal induction of other cytokines involved in the Th17 response (IL-23, IL-6, IL-1β, IL-27) were not inhibited by IAV (Fig 3D).

### Influenza A virus infection specifically targets TLR9 pathway in human monocytes

**Fig 4:**
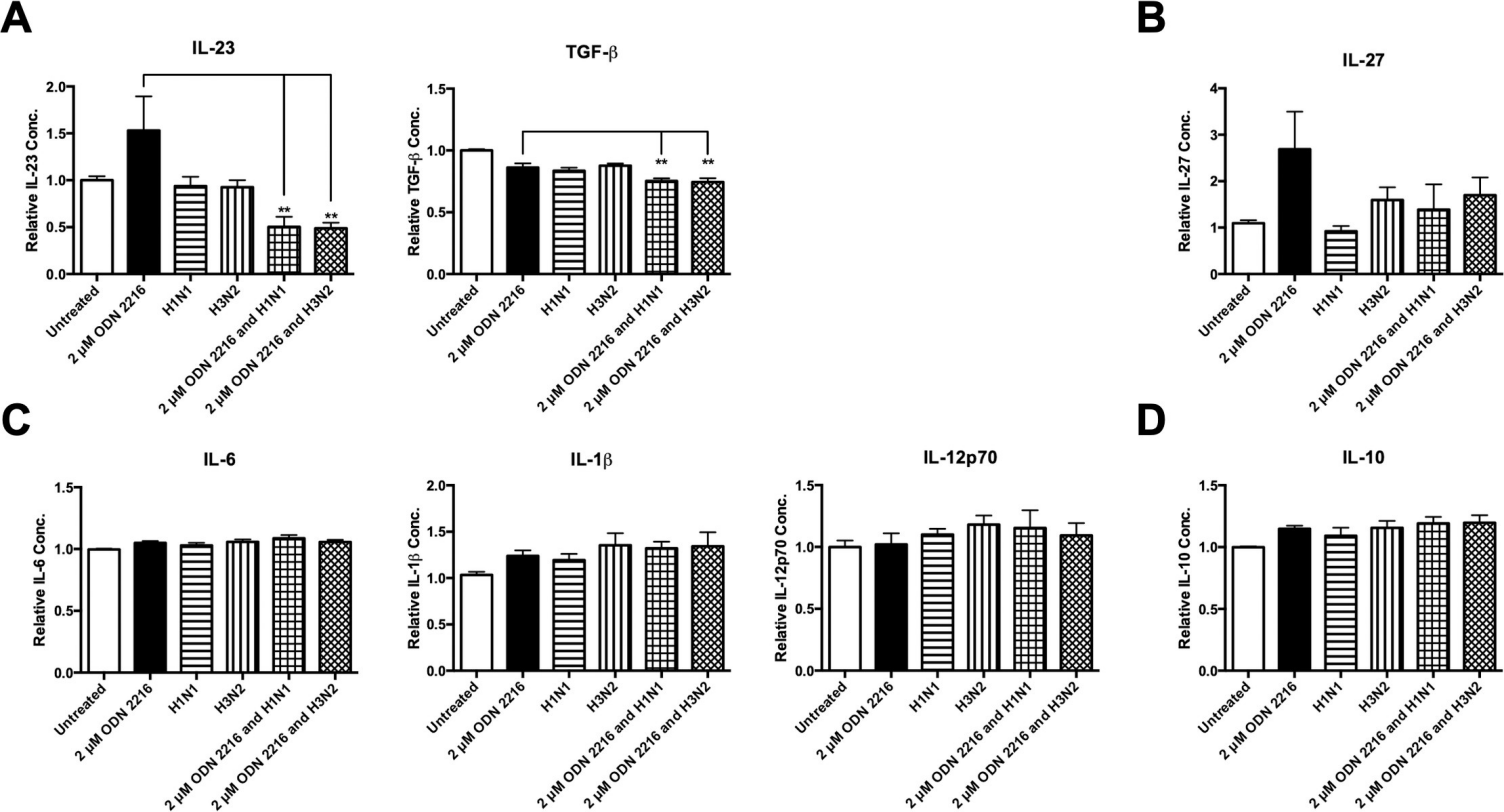
Live IAV specifically targets TLR9 pathway in human monocytes. Levels of (A) IL-23, TGF-β, (B) IL-27, (C) IL-6, IL-1β, IL-12p70, and (D) IL-10 secreted by CD14^+^ cells following 24 h treatment with a TLR9 mono-agonist (ODN 2216), live H1N1 or H3N2 alone or in combination with ODN 2216 (or untreated as a control) were determined by ELISA. Each column represents normalised mean cytokine levels + SEM of 3 technical repeats of each treatment in every donor. Each ELISA represents normalised results from multiple donors (n): (A) IL-23 (n = 9), TGF-β (n = 9), (B) IL-27 (n = 9), (C) IL-6 (n = 9), IL-1β (n = 9), IL-12p70 (n = 5), and (D) IL-10 (n = 9). Statistical analyses were performed to compare cytokine levels in cells treated with ODN 2216 versus cells exposed to live H1N1 or H3N2 in combination with ODN 2216 by fitting a One-Way ANOVA to the data and using a Sidak test to adjust for multiple testing (**p<0.01).

It has previously been reported in mice that specific ligands for TLR9 induced protection against influenza, and that TLR9 plays a protective role in the early stages of *S*. *pneumoniae* infection [44,45]. We used a TLR9 mono-agonist (ODN 2216) to stimulate cells to determine if IAV influences TLR9 agonism in primary human monocytes. We demonstrated that IAV infection inhibited ODN 2216 induced-IL-23 in human monocytes. Both H1N1 and H3N2 inhibited ODN 2216 induction of IL-23 (n = 9) and further reduced basal induction of TGF-β despite ODN 2216 treatment (n = 9) (Fig 4A). There was a reduction of ODN 2216-induced IL-27 by IAV, although this was not statistically significant (n = 9) (Fig 4B). There was no inhibition of IL-6 and IL-1β or IL-12p70 (n = 9) (Fig 4C). There was no increase in the anti-inflammatory cytokine, IL-10 (n = 9) (Fig 4D).

### Influenza A virus infection specifically targets TLR9-induction of RORC in human monocytes

**Fig 5:**
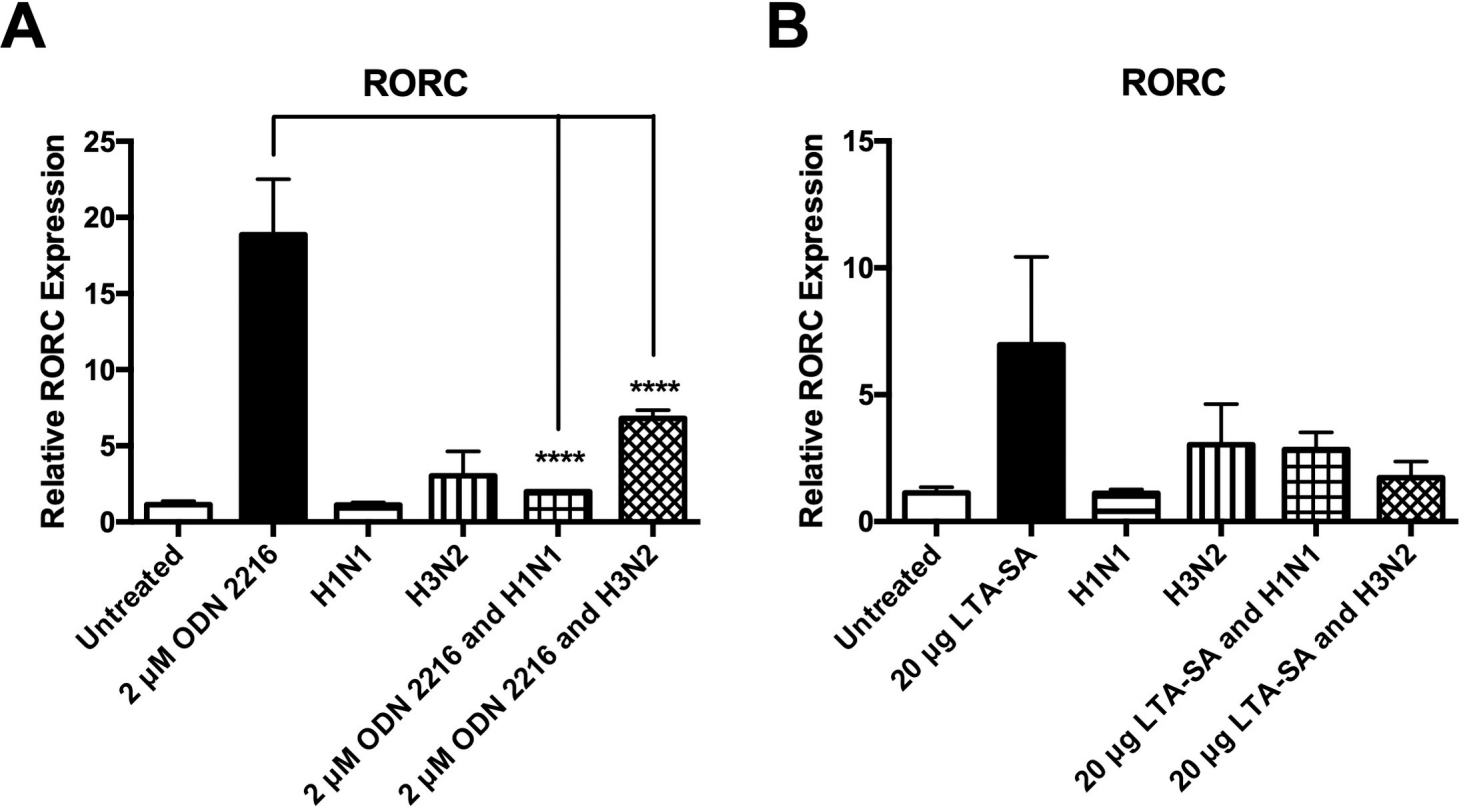
Live IAV specifically inhibits TLR9-induced RORC expression in human monocytes. Levels of RORC mRNA expression in CD14^+^ cells following 24 hr treatment with a (A) TLR9 or (B) TLR2 mono-agonist, (A & B) live H1N1 or H3N2 alone or in combination with each mono-agonist (or untreated as a control) were determined by qPCR. Each column represents mean amplification of gene of interest normalised to the mean expression of the reference gene, GAPDH (n = 3) + SEM (n = 3). Statistical analyses were performed to compare RORC mRNA expression in cells treated with the chosen agonist versus cells exposed to live H1N1 or H3N2 in combination with the chosen agonist by fitting a One-Way ANOVA to the data and using a Sidak test to adjust for multiple testing (****p<0.0001).

RORC is a transcription factor specific to the Th17 response, which plays an important role in the upregulation of IL-23 expression [17,18,20,46,47]. As TLR2 agonist and TLR9 agonist induction of IL-23 has been inhibited by IAV, we sought to investigate if this was due to IAV targeting expression of RORC in human monocytes. The RORC gene encodes for two protein isoforms (RORγ and RORγt), therefore the assay chosen targeted both variants. We have determined that IAV inhibits TLR9-induction of RORC (Fig 5A), however IAV does not inhibit TLR2-induction of RORC (Fig 5B).

### Influenza A virus infection does not target TLR5 pathway in human monocytes

**Fig 6:**
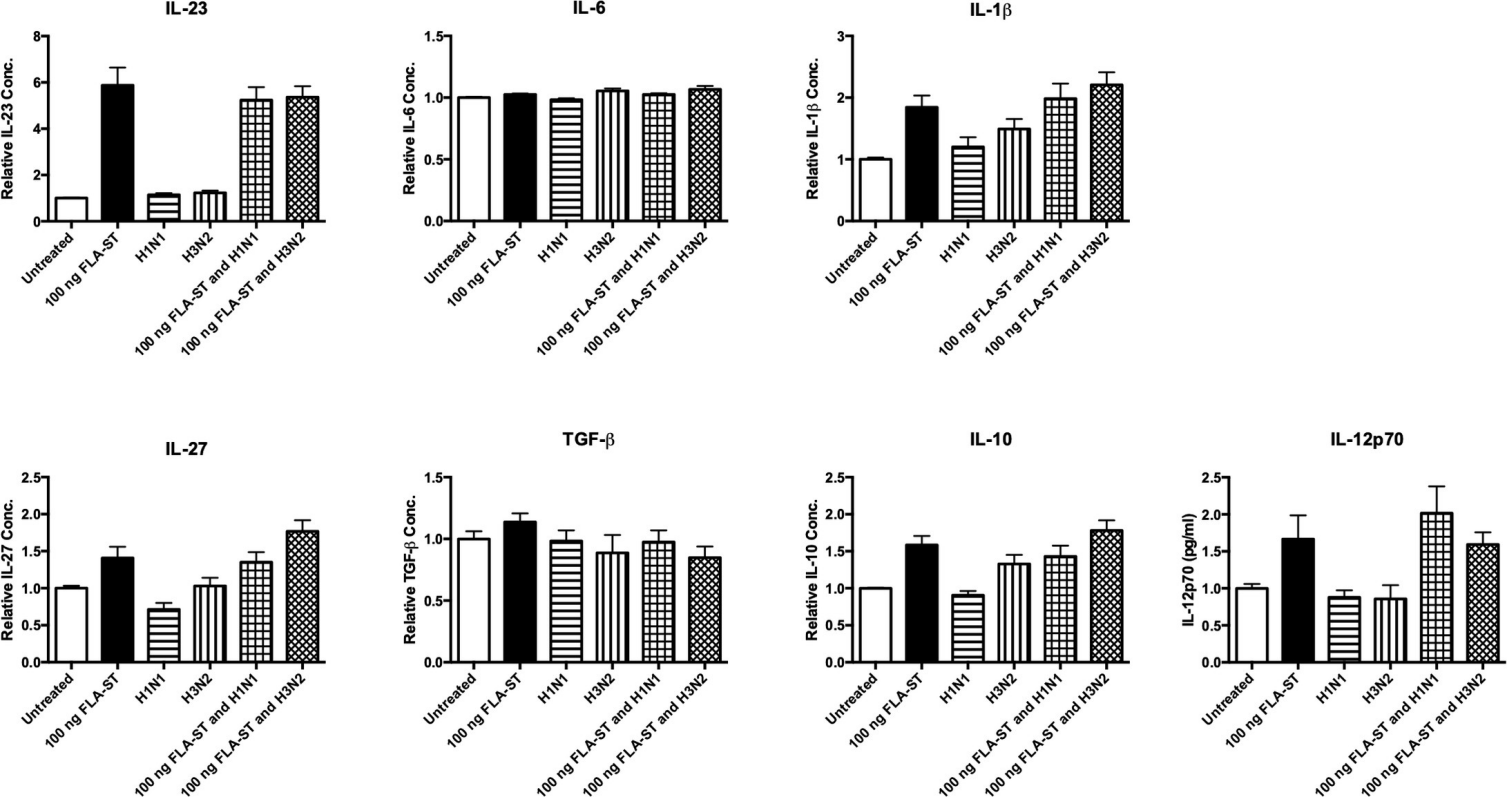
Live IAV does not target TLR5 pathway in human monocytes. Levels of IL-23, IL-6, IL-1β, IL-27, TGF-β, IL-10, and IL-12p70 secreted by CD14^+^ cells following 24 h treatment with a TLR5 mono-agonist (FLA-ST), live H1N1 or H3N2 alone or in combination with FLA-ST (or untreated as a control) were determined by ELISA. Each column represents normalised mean cytokine levels + SEM of 3 technical repeats of each treatment in every donor. Each ELISA represents normalised results from multiple donors (n): IL-23, IL-6, IL-1β, IL-27, TGF-β, IL-10, and IL-12p70 (n = 5). Statistical analyses were performed to compare cytokine levels in cells treated with FLA-ST versus cells exposed to live H1N1 or H3N2 in combination with FLA-ST by fitting a One-Way ANOVA to the data and using a Sidak test to adjust for multiple testing.

A TLR5 agonist has previously been shown to improve the efficacy of antibiotics in the treatment of IAV and *S*. *pneumoniae* co-infections in mice [34]. We used a TLR5 mono-agonist (FLA-ST) to stimulate cells and sought to determine if IAV had an effect on TLR5 agonsim in primary human monocytes. We have demonstrated that IAV does not inhibit FLA-ST induced cytokines (IL-23, IL-6, IL-1β, IL-27, TGF-β, IL-10, IL-12p70) (n = 5) (Fig 6).

### Treatment with a TLR5 mono-agonist restores inhibited immune responses to *S*. *pneumoniae* during influenza infection in human monocytes

**Fig 7:**
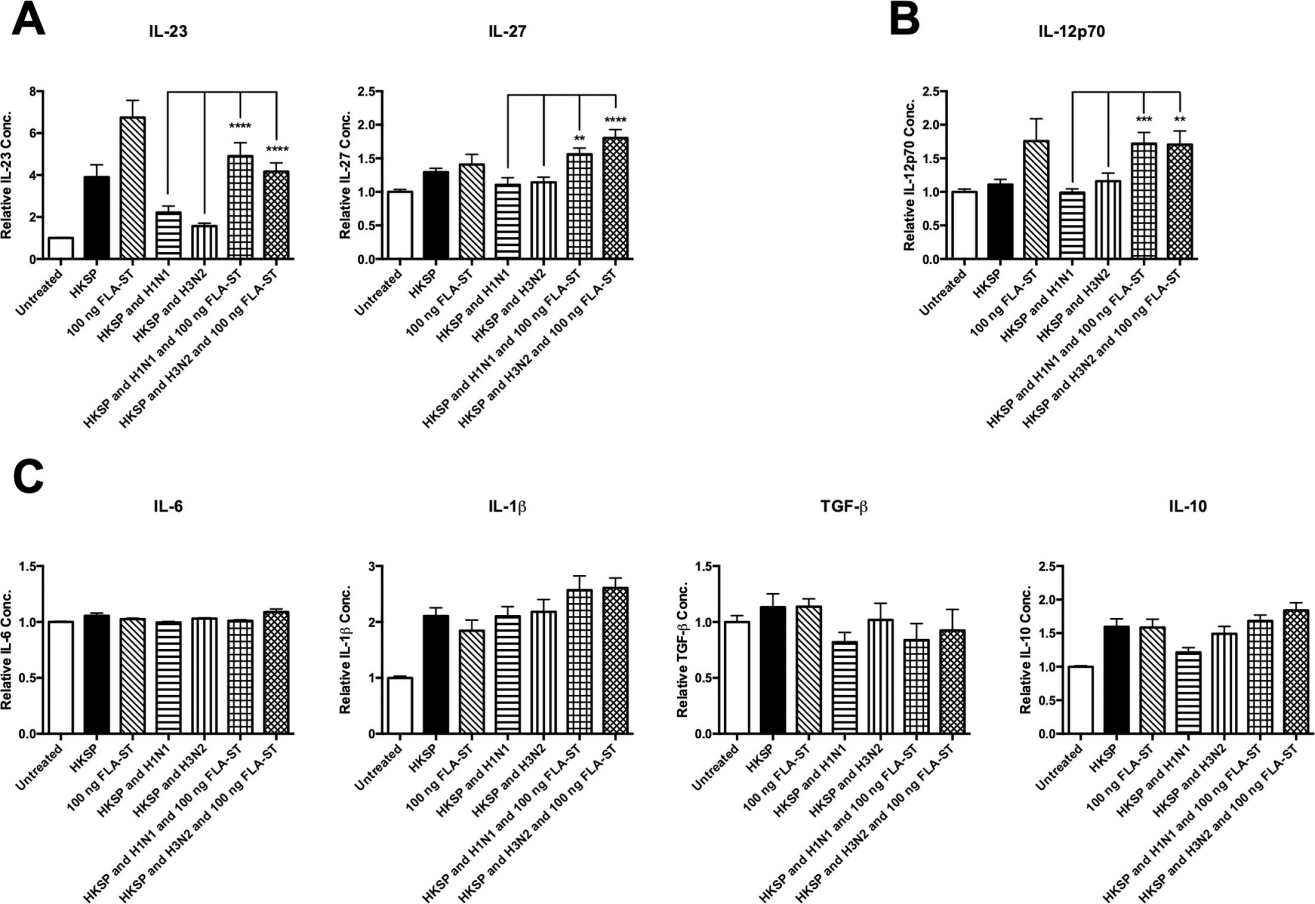
TLR5 agonism restores inhibited immune responses to *S*. *pneumoniae* during influenza infection in human monocytes. Levels of (A) IL-23, IL-27, (B) IL-12p70, (C) IL-6, IL-1β, TGF-β, and IL-10 secreted by CD14^+^ cells following 24 h treatment with HKSP, live H1N1 or H3N2 in combination with HKSP, live H1N1 or H3N2 in combination with HKSP and FLA-ST (or untreated as a control) were determined by ELISA. Each column represents normalised mean cytokine levels + SEM of 3 technical repeats of each treatment in every donor. Each ELISA represents normalised results from multiple donors (n): (A) IL-23 (n = 5), IL-27 (n = 5), (B) IL-12p70 (n = 5), (C) IL-6 (n = 5), IL-1β (n = 5), TGF-β (n = 5), and IL-10 (n = 5). Statistical analyses were performed to compare cytokine levels in cells exposed to live H1N1 or H3N2 in combination with HKSP versus cells exposed to live H1N1 or H3N2 in combination with both HKSP and FLA-ST by fitting a One-Way ANOVA to the data and using a Sidak test to adjust for multiple testing (**p<0.01, ***p<0.001, ****p<0.0001).

Having established that TLR5 mono-agonism is not inhibited by IAV, we sought to establish if this agonist could be used to restore previously observed inhibition of *S*.*p*-induced cytokines by IAV [14]. We demonstrated that TLR5 stimulation restored IAV-inhibited *S*.*p*-induced IL-23 and IL-27 (n = 5) (Fig 7A). Stimulation with FLA-ST increased IAV-*S*. *pneumoniae* IL-12p70 levels to above those observed in *S*. *pneumoniae* treated cells alone (n = 5) (Fig 7B). TLR5 mono-agonist treatment did not have any effect on levels of IAV-*S*. *pneumoniae* IL-6, IL-1β, TGF-β, and IL-10 (n = 5) (Fig 7C).

## Discussion

Secondary bacterial infections, such as those caused by *S*. *pneumoniae* are a major complication of IAV infection worldwide [1–7]. Th17 cells are key in clearing *S*. *pneumoniae* infections from the lung, however studies have revealed that IAV infection inhibits the Th17 response in mice and human monocytes [12–14]. Here, we aim to establish if IAV specifically targets *S*.*p-* associated TLRs. The TLRs involved in detecting *S*. *pneumoniae* infection are TLR2, TLR4, and TLR9 [16]. Therefore, we examined the effects of IAV on TLR mono-agonist induction of cytokines involved in the Th17 response to in primary human monocytes. We have shown that IAV targets aspects of each of the *S*. *pneumoniae* associated TLR pathways.

We initially examined the levels of viral NP across treatments and donors. Levels of viral NP did not differ between cells infected with IAV and those co-treated with IAV and *S*. *pneumoniae* (Fig 1). Additionally, levels of NP were very similar across different donors, indicating very reproducible infectivity by IAV. Differences in infectivity between strains maybe due to H1N1 being a laboratory-generated virus, whereas H3N2 is a clinical isolate.

We have demonstrated that the TLR2 and TLR9 pathways appear to be inhibited more strongly by IAV than the TLR4 pathway. However, the TLR4 mono-agonist was not as strong of an inducer of these cytokines. In addition, there are slight variations in the signalling pathways of these receptors. Both the TLR2 and TLR9 pathways require recruitment of the adaptor molecule MyD88 to trigger downstream signalling [48–51], whereas the TLR4 pathway can recruit MyD88 in addition to a different adaptor molecule, TRIF to trigger downstream signalling [49,50]. As TLR4 has the ability to signal through two distinct pathways, this may be why TLR4 agonism appears to be less susceptible to inhibition by IAV. TLR2- and TLR9-induced IL-23 is inhibited by both strains of IAV (Figs 2A and 4A). This inhibition of IL-23 is interesting as we have previously shown that IAV inhibits whole *S*. *pneumoniae*-induced IL-23 [14]. IL-23 is necessary for the expansion and commitment to the expression of the Th17 lineage, in particular the cytokine IL-17A, which is pivotal in the response to *S*. *pneumoniae* [52,53].

Both H1N1 and H3N2 inhibited TGF-β induction during TLR agonist treatment. TGF-β is a multifunctional cytokine which exhibits both pro-inflammatory and anti-inflammatory properties [54,55]. This cytokine is involved in the differentiation of both Th17 cells and Treg cells in a concentration-dependent manner [56]. At low concentrations, TGF-β synergises with IL-6 to promote Th17 expression and at high concentrations, TGF-β represses Th17 expression in favour of Treg expression [56]. As TLR-induced TGF-β has been inhibited by both strains of IAV (See Figs 2A, 3A and 4A), it may have a negative effect on Th17 differentiation. It is interesting that TLR agonism of TGF-β was inhibited by IAV, and yet in a previous study, whole *S*. *pneumoniae*-induction of TGF-β was not inhibited by IAV [14]. It is possible that whole *S*. *pneumoniae* contains components that activate pathways related to TGF-β which IAV either cannot or does not target. As different TLRs often dimerise with each other [57], it may be that IAV can target individual (mono)TLR activation of TGF-β, but that it cannot exert the same inhibitory effects on TLRs when in heterodimer conformation (which is likely with whole *S*. *pneumoniae*). It should be noted that IL-6, the cytokine with which TGF-β synergises has not been affected in any way as it has neither been strongly induced by any of the TLR mono-agonists or inhibited by IAV likewise. It may be that IL-6 is not induced by mono-agonists. It may require heterodimer activation in order to be induced above a basal level. TLR-induced IL-6, IL-1β, IL-27, and IL-12p70 (Figs 2C and 2D, 3B and 3D and 4C and 4D) were not affected by IAV, thus suggesting that IAV is having a specific effect on TLR-induced TGF-β and IL-23. There have been numerous other studies which have reported that IL-1β is induced by IAV in humans and mice [58–60], although such induction occurred at later time points in these studies. Considering such phenomena, the lack of inhibition of IL-1β by IAV is unsurprising. There are numerous pathways whereby IL-1β is induced, which may be why IL-1β is not inhibited by IAV as with other Th17 cytokines [61].

As IAV inhibited TLR2 and TLR9 induction of IL-23, this cytokine was researched further to determine why IL-23 specifically may be targeted by IAV. IL-23 signals through IL-23R and IL-12Rβ1, which leads to phosphorylation of STAT3 [46]. Phosphorylation of STAT3 leads to induction of RORC, which is a Th17-specific transcription factor. RORC in turn, induces further expression of IL-23R, which increases IL-23 cytokine induction in a positive-feedback loop [17,46,47,62,63]. As RORC has been shown to be important in the induction of IL-23, we sought to determine if RORC induction by both TLR2 and TLR9 was inhibited by IAV. We have demonstrated that TLR2 agonist induction of RORC was not significantly inhibited by IAV (Fig 5B). Indeed, TLR2 agonist induction of RORC was inconsistent, with one donor inducing very high levels of RORC in response to TLR2 agonism, whereas two donors did not induce RORC past basal levels. RORC has been shown to be induced at different time points, therefore, it is possible that induction of RORC in response to TLR2 agonism is peaking at an earlier time [28]. Additionally, we have demonstrated that TLR9 agonist induction of RORC is significantly inhibited by IAV (Fig 5A). The inhibition of RORC is most likely why TLR9-induction of IL-23 is inhibited, and may also be why *S*. *pneumoniae* induction of IL-23 was inhibited previously [14]. As RORC expression is induced by phosphorylation of STAT3, and inhibited by phosphorylation of STAT1, the effect of IAV on STAT3 and STAT1 phosphorylation may shed further light on the mechanisms behind IAV inhibition of *S*. *pneumoniae* and associated TLR responses. IAV-mediated inhibition of TLR-induction of RORC has not been demonstrated in human monocytes previously.

TLR5 agonism has been shown to elicit protection in mice against multiple bacterial pathogens including *Clostridium difficile*, and vancomycin-resistant *Enterococcus*, and *S*. *pneumoniae* [64–66]. Additionally TLR5 agonism has improved the efficacy of antibiotics in treating IAV and *S*. *pneumoniae* co-infections in mice [34]. TLR5 is activated in response to flagellin [67], and therefore is not activated in response to *S*. *pneumoniae* infections. Due to the encouraging results using TLR5 agonism as a treatment in mice, we sought to investigate what effect TLR5 agonism may have on immune responses *S*. *pneumoniae* and IAV co-infection by human monocytes.

We have established that the TLR5 pathway is not targeted by IAV as cytokine secretion is not impaired by IAV (Fig 6). Indeed, IL-23 is induced robustly by a TLR5 mono-agonist, even in the presence of IAV. Although, induction of TGF-β by the TLR5 mono-agonist, FLA-ST was decreased during IAV infection, this reduction was not statistically significant. A recent study in mice demonstrated that administration of a TLR5 agonist during IAV infection resulted in a decrease of viral RNA [68]. This may explain why IAV infection does not inhibit TLR5 mono-agonist induced cytokines. As this pathway is not affected by IAV and to address the gap in the research using human models, we examined TLR5 agonism in a simulated co-infection with IAV and *S*. *pneumoniae* in human monocytes. TLR5 agonism restored IAV-inhibited levels of *S*.*p*-induced IL-23 and IL-27 (Fig 7A), and induced level of IL-12p70 to above those observed in *S*. *pneumoniae* treated cells alone (Fig 7B).

Results in mouse models have been very promising, however due to differences in fundamental immune responses between mice and humans, it is imperative that similar investigations be carried out in human models. Thus far, the effect of IAV infection on *S*. *pneumoniae* associated TLRs has not been examined in human monocytes. The results presented in this human ex vivo model demonstrate novel insights into the effect of influenza on crucial *S*. *pneumoniae*-associated TLRs, and corroborates and provides mechanistic insights into the beneficial effects of TLR5 in mouse models. Additionally, this is the first study to demonstrate the inhibitory effect of IAV on the human Th17-specific transcription factor, RORC. This study highlights the importance of the treatment strategies that may be possible by utilising certain TLR pathways. However, caution must be used when considering the therapeutic benefits of restoring Th17 responses as an increase in the cytokine IL-23 and other Th17 polarising cytokines have been associated with pathogenesis and the development of inflammation in a range of autoimmune diseases such as rheumatoid arthritis, psoriasis, and Crohn’s disease [21,69–71]. Therefore, future treatments concerning targeting the Th17 pathway should be carefully considered.

## Supporting information

S1 Fig Statistical analyses of IL-23 secretion in Untreated vs TLR agonist-treated monocytes. A One-Way ANOVA was performed comparing the levels of IL-23 secretion between Untreated monocytes and TLR-agonist treated monocytes.
(TIFF)

S2 Fig Representative result for Fig 2. Representative donor displaying result for TLR2 agonism.
(TIFF)

S3 Fig Representative result for Fig 3. Representative donor displaying result for TLR4 agonism.
(TIFF)

S4 Fig Representative result for Fig 4. Representative donor displaying result for TLR9 agonism.
(TIFF)

S5 Fig Representative result for Fig 6. Representative donor displaying result for TLR5 agonism.
(TIFF)

S1 Data Numerical data underlying all figures. (XLSX)
