# Supplementary figures and images for: A TLR5 mono-agonist restores inhibited immune responses to Streptococcus pneumoniae during influenza virus infection in human monocytes
Source: PLoS One. 2021 Oct 13;16(10):e0258261. doi: 10.1371/journal.pone.0258261 (PMC8513880; doi:10.1371/journal.pone.0258261)

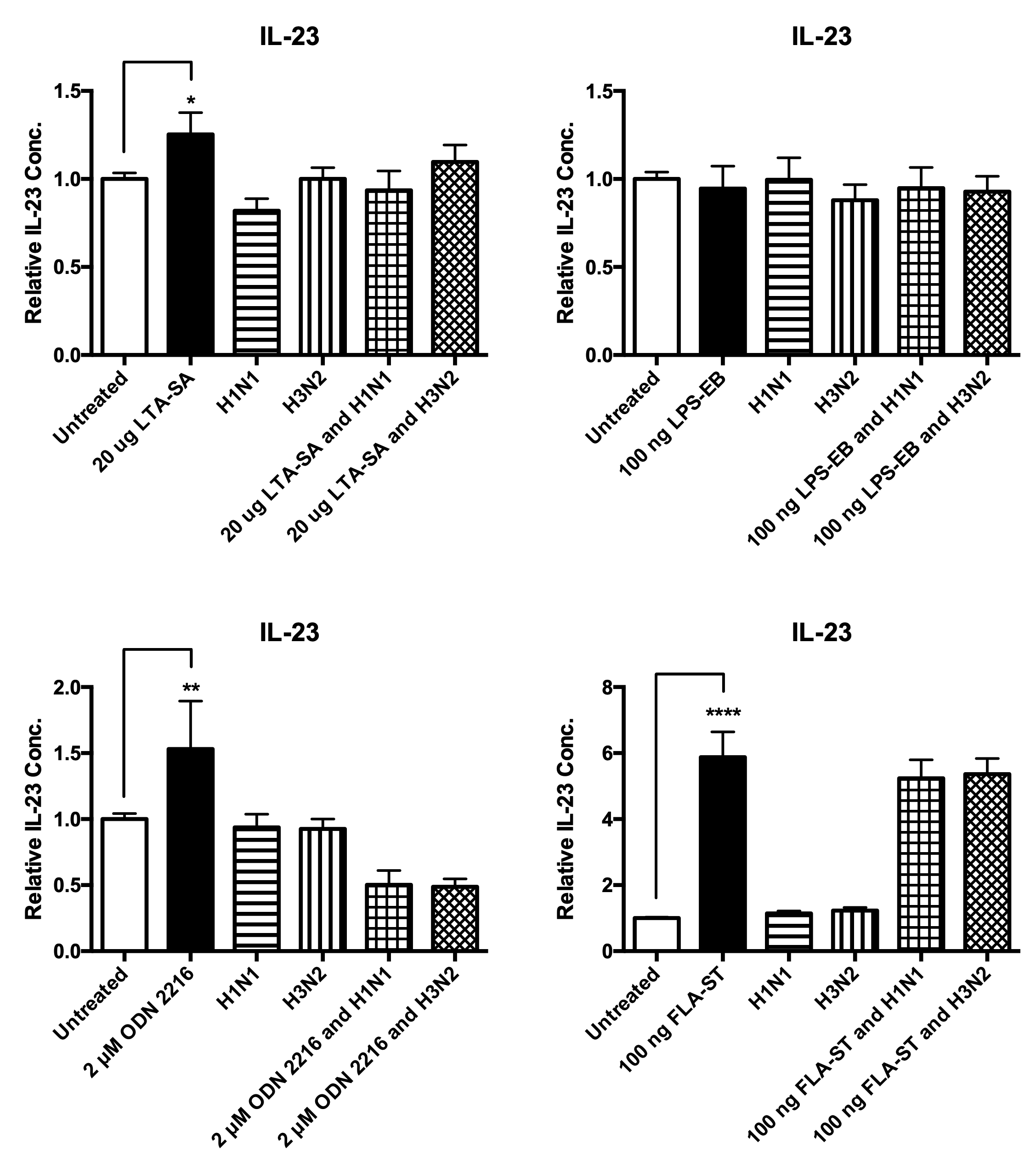

Supplement: S1 Fig — A One-Way ANOVA was performed comparing the levels of IL-23 secretion between Untreated monocytes and TLR-agonist treated monocytes. (TIFF) [file pone.0258261.s001.tiff]

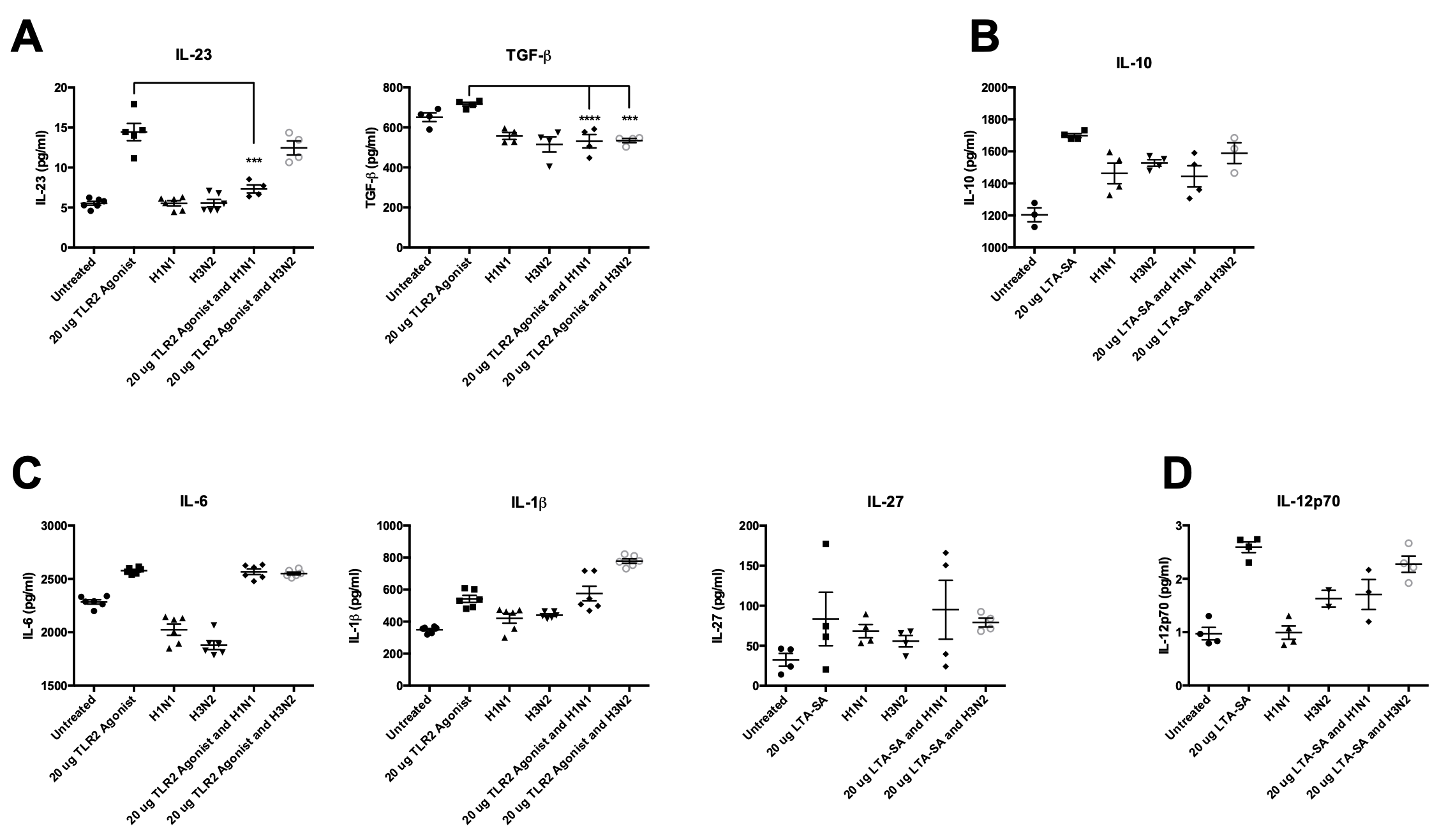

Supplement: S2 Fig — Representative donor displaying result for TLR2 agonism. (TIFF) [file pone.0258261.s002.tiff]

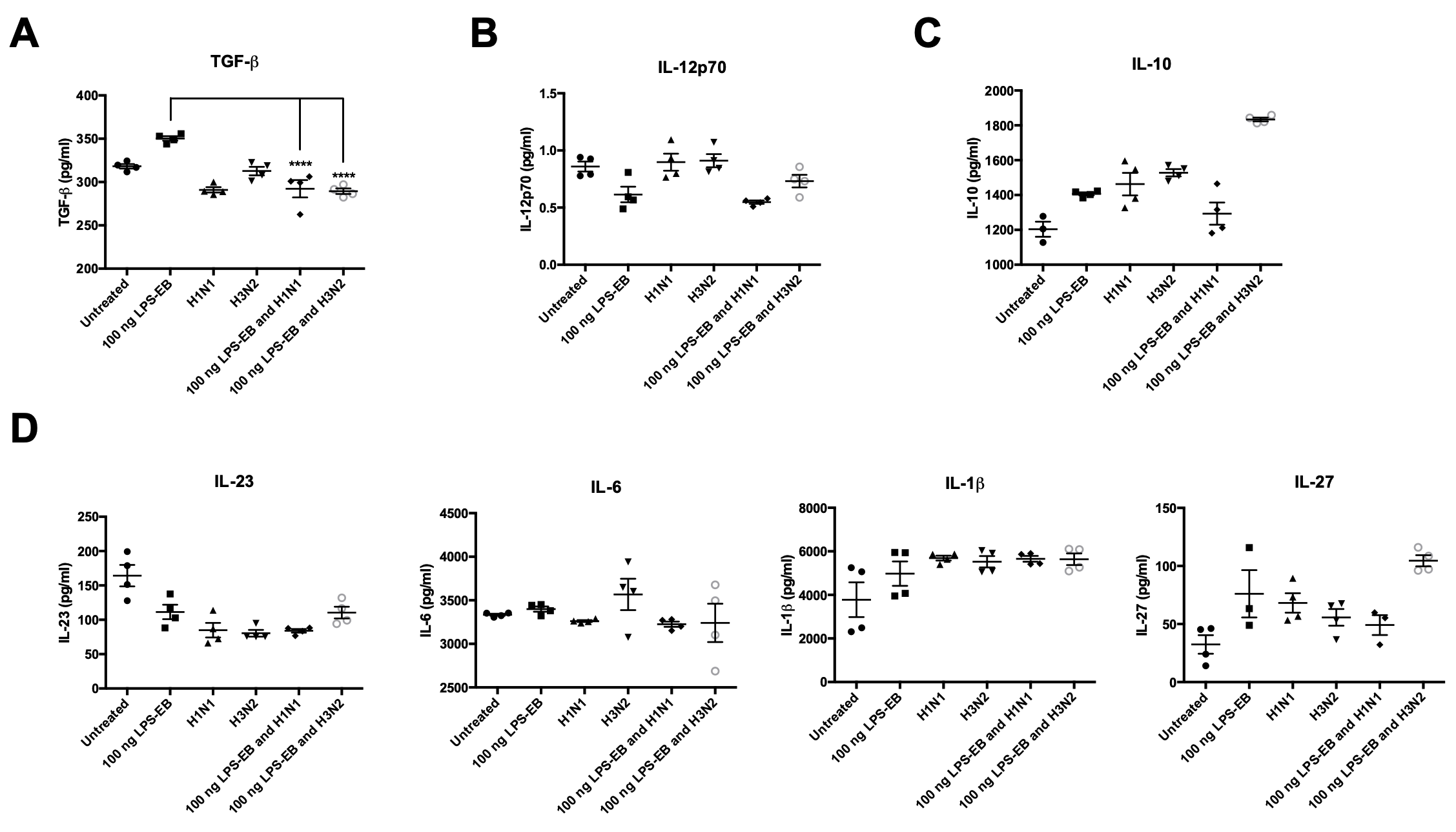

Supplement: S3 Fig — Representative donor displaying result for TLR4 agonism. (TIFF) [file pone.0258261.s003.tiff]

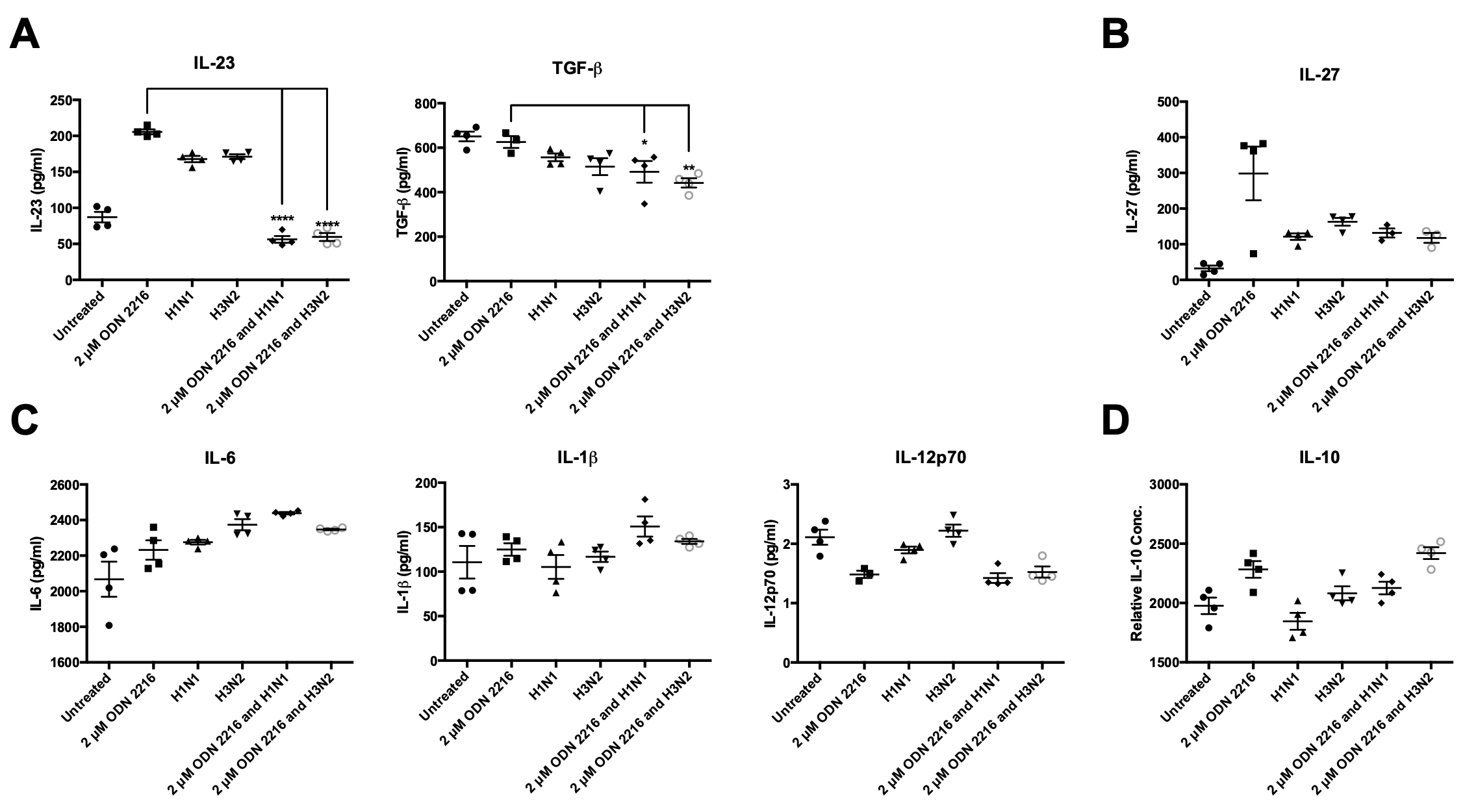

Supplement: S4 Fig — Representative donor displaying result for TLR9 agonism. (TIFF) [file pone.0258261.s004.tiff]

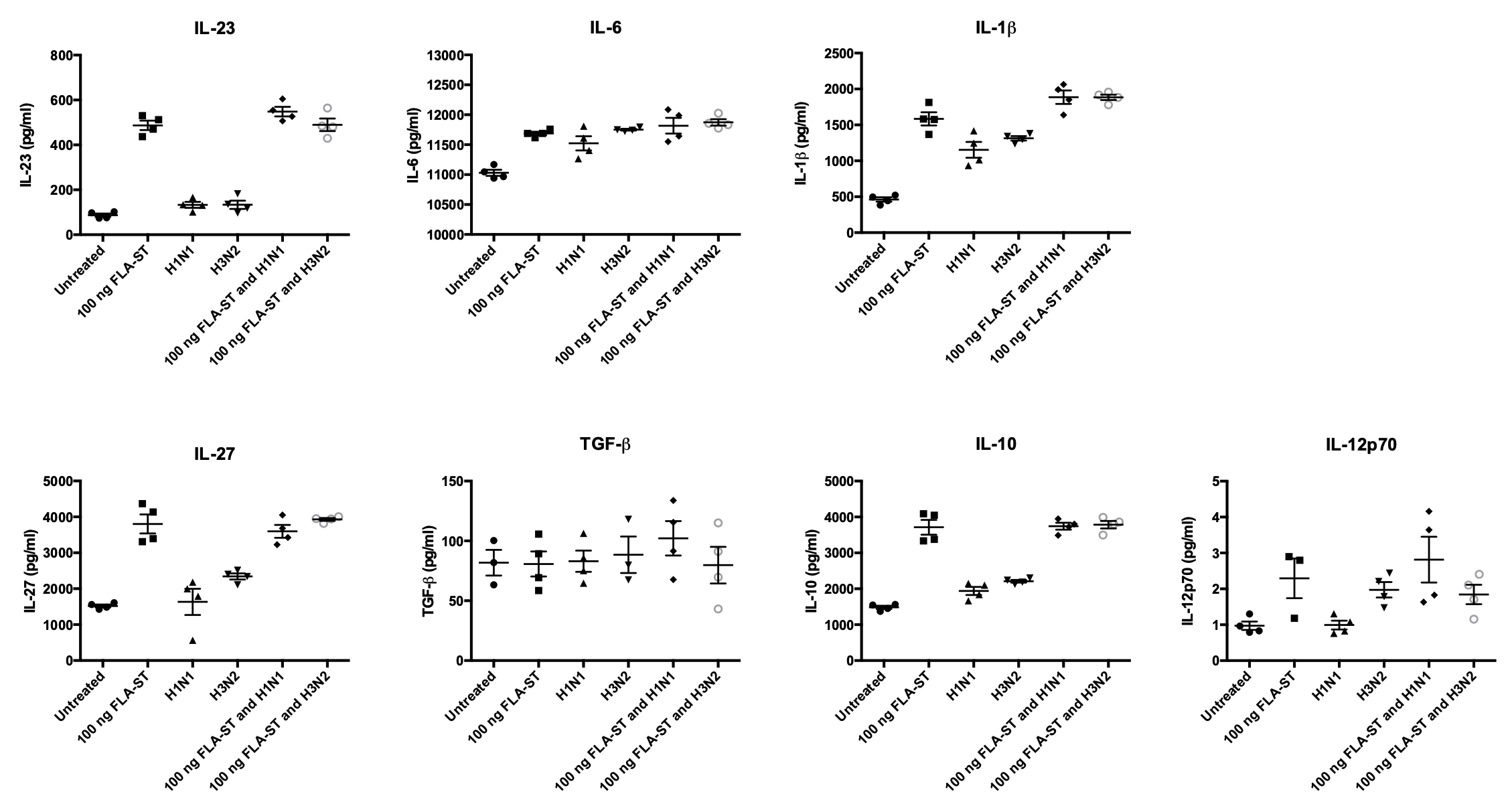

Supplement: S5 Fig — Representative donor displaying result for TLR5 agonism. (TIFF) [file pone.0258261.s005.tiff]
